# Translation and validation of the *Tinnitus Primary Function Questionnaire* into Brazilian Portuguese

**DOI:** 10.1016/j.bjorl.2022.06.004

**Published:** 2022-07-05

**Authors:** Patrícia Perez Coradini, Sabrina Nuñes Gonçalves, Jeanne Oiticica

**Affiliations:** a Universidade de São Paulo, Programa de Graduação em Otorrinolaringologia, São Paulo, SP, Brazil; b Universidade Federal do Rio Grande do Sul, Programa de Graduação em Saúde da Criança e do Adolescente, Porto Alegre, RS, Brazil

**Keywords:** Tinnitus, Quality of life, Validation study, Questionnaire

## Abstract

**Objective:**

In this study, the Tinnitus Primary Function Questionnaire was translated, culturally adapted and validated for Brazilian Portuguese.

**Methods:**

This study was carried out in two stages. First, a prospective study of translation and cultural adaptation was carried out with a group of 20 patients. The questionnaire was translated, revised and back-translated from Portuguese into English. Subsequently, a retrospective study was carried out with 1,095 patients, 18 months after the first stage. The validation and reliability of the Tinnitus Primary Function Questionnaire was verified by comparing its results with the results of the Tinnitus Handicap Inventory.

**Results:**

The interclass correlation coefficient of the behavioral aspects ranged from 0.82 to 0.90 and the total score was 0.93, while Cronbach’s alpha was >0.94 for the total score during the test-retest application. To validate the questionnaire, a database was used, which consisted of 1,095 patients submitted to tinnitus assessment using the Tinnitus Primary Function Questionnaire and Tinnitus Handicap Inventory simultaneously. Patients were enrolled from 14 Brazilian states (46% female), with a mean age of 58 years. The reliability of the Tinnitus Primary Function Questionnaire was tested during validation for the four different evaluated behavioral aspects, and the results were significantly high for all aspects and the total score. To validate the Tinnitus Primary Function Questionnaire, the results of the total Tinnitus Primary Function Questionnaire and Tinnitus Handicap Inventory scores were compared using Pearson’s product-moment correlation test. The results indicate a high correlation between the two questionnaires (r = 0.84; *p* < 0.001; 95% CI 0.82–0.85).

**Conclusion:**

The data from the Tinnitus Primary Function Questionnaire showed a high correlation with those of the Tinnitus Handicap Inventory. This indicates that the Portuguese version of the Tinnitus Primary Function Questionnaire can be adopted as a valuable tool in the clinical evaluation of patients with tinnitus.

**Level of Evidence:**

2C.

## Highlights

• The translated version of the Tinnitus Primary Function Questionnaire showed good test-retest accuracy with robust interclass correlation coefficient values.
• The Brazilian version of the Tinnitus Primary Function Questionnaire showed high reliability according to the results of Cronbach’s alpha.
• Pearson’s correlation coefficient demonstrated a strong correlation between the total scores of Tinnitus Primary Function Questionnaire and Tinnitus Handicap Inventory scores.

## Introduction

Tinnitus can be described as an auditory sensation, without an external sound stimulus, which can manifest itself through unpleasant experiences and impact patient quality of life.^1^ Researches indicate that tinnitus is a prevalent symptom; affecting 10%–15% of the world’s population,^2^ and that in Brazil, in the city of São Paulo, its prevalence is about 22%.^3^ Approximately 20% of people who experience tinnitus require clinical intervention.^4^

Tinnitus is not a specific disease, but rather a symptom that can be associated with multiple diseases.^4^ Considering its high incidence and the multifactorial aspect of this condition, questionnaires have been increasingly adopted, not only for the initial diagnosis, but also as a tool for clinical follow-up of patients undergoing treatment, aiming at quantifying the actual impact of the tinnitus on their quality of life.^5^, ^6^, ^7^, ^8^, ^9^, ^10^, ^11^

It is important to emphasize, however, that although there are several available questionnaires in the literature to evaluate the impact of tinnitus, it is necessary to understand the actual objective of each instrument, when it was developed and its validation process. Thus, choices can be made according to the desired objective. Some questionnaires quantify the impact of tinnitus based on quality of life, others provide specific information on the most affected behavioral aspects, whereas some address the follow-up of the clinical outcome. Therefore, a real understanding of the purpose of the created instrument, and its validation process, is very important for choosing the questionnaire to be used.^12^

However, until recently, there was only one available questionnaire in Brazil, the Tinnitus Handicap Inventory (THI).^13^ This questionnaire shows high clinical applicability, being very useful for measuring tinnitus severity. However, results regarding behavioral areas of greatest impact on the patients’ quality of life are quite limited. Therefore, a gap remains regarding the measurement of this aspect, that could be very useful, especially when counseling patients who suffer from tinnitus. In 2021, the validation into Brazilian Portuguese of the Tinnitus Function Index Questionnaire (TFI) which assesses tinnitus in eight subscales, was published.^14^ The main purpose of the TFI questionnaire is to determine symptom severity, in addition to monitoring pre- and post-therapeutic intervention clinical outcome.^15^, ^16^

In the present study, the Iowa Tinnitus Activities Questionnaire (TAQ), also known as Tinnitus Primary Function Questionnaire (TPFQ) was translated and validated into Brazilian Portuguese and used to measure tinnitus severity and assist in patient counseling. The TPFQ has already been internationally validated in several languages.^17^, ^18^, ^19^, ^20^, ^21^, ^22^, ^23^, ^24^ There is a clear foundation of what the TPFQ seeks to assess, particularly related to the measurement and targeting of the Tinnitus Activities Treatment (TAT), which comprises structured counseling to be used alone or associated with sound therapy.

The TPFQ questionnaire includes affirmative sentences related to daily activities, which patients need to quantify in relation to the impact that each statement has on their quality of life. The obtained score helps to understand the patient’s reaction to tinnitus.

Overall, the validation of a questionnaire includes measures of internal consistency, which allows verifying whether all subparts of the instrument measure different aspects of the same characteristic. The validation, in turn, can be carried out by comparing the result of the questionnaire to be validated to another that is already well known and accepted in the language to be applied.^25^, ^26^, ^27^, ^28^

In the medical literature, the most translated and used questionnaire is the THI, that provides information about the general level of discomfort using the total score, which is divided into three domains: functional, emotional and catastrophic. This questionnaire is widely used because it is short and easy to apply. However, it does not provide information related to life activities impacted by tinnitus, which are very useful to treat the reaction and the impact of tinnitus on patient quality of life.^21^

In 2003, Tyler et al. developed the Iowa Tinnitus Activities Questionnaire (TAQ), a preliminary version of the TPFQ, validated in 2014.^29^ The TAQ was developed to assess how tinnitus impacts the patients’ quality of life. The aim was to provide a sensitive tool to measure clinical changes and highlight areas in which patients can benefit from counseling to manage their reaction to tinnitus. The TPFQ, in turn, provides clues about the severity and impact of tinnitus on essential daily activities.^29^, ^30^, ^31^ In the TPFQ validation, it was observed that the questionnaire has high internal consistency in relation to the total score and to all evaluated behavioral aspects: sleep, hearing, concentration and emotion, proving to be a sensitive instrument to assess the impact of tinnitus on quality of life.

The TPFQ, developed by Tyler et al.,^29^ is used to guide the direction of tinnitus counseling, as the therapist can prioritize and focus on the high-scoring aspects of the questionnaire. Therefore, patients who scored higher on the questionnaire regarding the sleep aspect, for instance, would have different counseling and rehabilitation for tinnitus when compared to those with higher scores in the concentration aspect.^29^

While several questionnaires for tinnitus assessment are available in English, very few are available in Brazilian Portuguese. The THI was developed in 1996 by Newman et al.^32^; in 2005, it was translated and culturally adapted into Brazilian Portuguese by Ferreira et al.^32^ and validated for Brazilian Portuguese, in 2006, by Schmidt et al.^13^ Recently, Rosa et al.^14^ validated the Tinnitus Functional Index Questionnaire (TFI), which assesses tinnitus in eight subscales. The main purpose of the TFI questionnaire is to determine the severity of the symptom, in addition to monitoring pre- and post-therapeutic intervention clinical outcome.^14^ However, given the high prevalence of tinnitus in the Brazilian population,^3^ it is essential to have other tools to assess the severity of tinnitus and guide the best treatment and counseling approach. Therefore, in the present study, the TPFQ was submitted to translation, cultural adaptation and validation into Brazilian Portuguese.

The translation of the TPFQ followed the full process of translation and cultural adaptation according to the methodology described by Guillemin et al.^33^ and by Terwee et al.^34^ for the creation of the Brazilian version. This included the translation, review and cross-cultural adaptation, and back-translation of the questionnaire. Then, the Brazilian version of the TPFQ was validated by comparing its total score with the total score of the THI, by calculating Pearson’s correlation coefficient.^34^

## Methods

The use of the TPFQ questionnaire in this study was previously authorized, via electronic means, by Professor Dr. Richard S. Tyler, the author of the original questionnaire in the English language. The patients with chronic tinnitus selected for the present study were registered in the database of private institutions that operate in different states of Brazil. The study was carried out in two stages. The first stage was prospective, with the translation and cultural adaptation of the TPFQ. The second stage was retrospective, carried out 18 months after the completion of the first stage.

The first stage sample consisted of 20 individuals, corresponding to approximately 10% of the sample calculation for validation. The literature recommends that, before carrying out a validation of a questionnaire, it should be applied to a small group of people to verify whether the text translated into the desired language is understandable.^33^, ^35^, ^36^ During the application of the questionnaires, items that generated difficulty in understanding, went through subsequent percentage analysis. In case of a percentage value greater than 20%, the question had to be reviewed by the translation review group and applied again to the entire sample.^32^

The second stage corresponded to the validation of the TPFQ, carried out through criterion validity, comparing it with the THI. According to Terwee et al.,^34^ for each item of the questionnaire to be validated, 10 patients should be counted. As the TPFQ has 20 items, the second stage should have a sample of 200 patients or more. The database used in the present study had a much larger sample and, for this reason, a total of 1,095 patients was included, aiming to obtain more accurate estimates of validity and reliability.

### Translation and cultural adaptation

**Figure 1:**
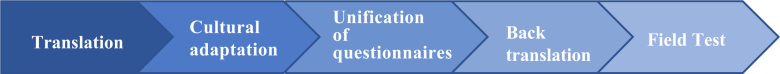
Stages of the TPFQ translation and cultural adaptation process.

The translation and cultural adaptation was carried out with 20 patients who were registered in the database and had complaints of chronic tinnitus and did not adhere to the tinnitus treatment before this research was carried out. All of them signed the free and informed consent form. The process was carried out (Fig. 1) as follows:

1 Translation: The English version of the TPFQ was distributed to three English translators-interpreters, who did not know each other or knew the content of the questionnaire. The objective was the individual and confidential creation of the first version of the questionnaire in Brazilian Portuguese. This procedure was intended to generate three independent translations (TPFQ-P1, TPFQ-P2, TPFQ-P3).
2 Review and cultural adaptation of the questionnaire: The review group, comprising professionals who had not been part of the previous translation process, consisted of an otorhinolaryngologist and two speech therapists – all Brazilians and proficient in English language. They analyzed the three documents resulting from the initial translations and reduced their differences. Subsequently, choices were made for each of the questions, regarding the best expressions and words, in addition to adapting the text to Brazilian cultural knowledge. Each reviewer provided a final version of the translated TPFQ questionnaire.
3 Unification of the TPFQ questionnaires: The researcher analyzed the final versions of the questionnaire resulting from each reviewer, noted whether there were differences between the versions and, if so, chose the option with the greatest consensus among the reviewers. After this analysis, the final unified version of the TPFQ questionnaire was created, translated and culturally adapted to Brazilian Portuguese.
4 Back-translation of the TPFQ questionnaire: After the unification of the questionnaires into a single version in Brazilian Portuguese, the back-translation was carried out. For this purpose, a copy was given to three other translators, who had not participated in the translation from English to Portuguese in the first stage. They performed the Portuguese-English back-translation. They also verified the agreement of the questions now back-translated with the original instrument in English. They found that there was agreement with the original version. Therefore, the final version of the TPFQ in Brazilian Portuguese was established.
5 Application of the TPFQ questionnaire: the reproducibility and precision of the TPFQ was evaluated through the application at two different moments, with an interval of seven to ten days between each application. In this phase, the items that generated difficulty for the patients to understand were listed, for later analysis in percentage values. If this percentage was greater than 20%, the question had to be reviewed by the review group and applied again to the entire sample.^32^

### TPFQ validation

The validation was performed after updating the TPFQ in the Brazilian Portuguese version, translated and culturally adapted in the first stage of this research in the care protocol of the private institution. The study was carried out by exporting the database, 18 months after the insertion of the TPFQ. Again, ethics related to the data and the commitment to preserve the subjects’ identity were respected.

The TPFQ criterion validity was performed by comparing it with the THI. The comparison was made by analyzing the total TPFQ score and the total THI score. The crossing of the quantitative variables obtained in the two instruments allowed the calculation of Pearson’s correlation coefficient (r) to analyze the positive correlation between the obtained results. The correlation of the TPFQ aspects (sleep, hearing, concentration, emotional) with the THI domains (emotional, functional, catastrophic) was also analyzed using the same coefficient (r).

### Statistical analysis

The collected data were recorded and exported to an Excel spreadsheet for further analysis. The value of *p* ≤ 0.05 was considered significant, and the analyses were performed using the software SPSS version 22.0.

During their application for the cultural adaptation, the questionnaires were applied to the same patients, at two different moments in time, aiming to analyze the test-retest accuracy. To quantitatively assess the variables, data from total scores and scores by behavioral aspects were used.

The interclass class correlation coefficient (ICC) calculations were performed to compare the results obtained in the first and second stages of application of the questionnaires. Values ​​closer to 1.0 indicate greater precision. In the present study, 0.70 was considered the minimum ideal to confirm an accurate test-retest result.^25^, ^26^, ^27^, ^28^ Cronbach’s alpha calculation was applied to measure the internal consistency of the questionnaires, which allows identifying the homogeneity of the items that comprise them. In this analysis, the value of 0.70 was also considered as the minimum to indicate adequate internal consistency of the data.^26^, ^28^ Data from the total scores were used to analyze the variables quantitatively and Cronbach’s alpha was calculated for the results of the 1st application (test) and the 2nd application (retest) of each of the questionnaires.

The validation of the TPFQ questionnaire was performed by analyzing its total score and the total THI score. The crossing of the quantitative variables obtained in the two instruments allowed the calculation of Pearson’s correlation coefficient (r). The correlation of the TPFQ aspects (sleep, hearing, concentration, emotion) with the THI domains (emotional, functional, catastrophic) was also analyzed using the same coefficient (r). Pearson’s correlation coefficient was used because it allows calculating the level of correlation between quantitative variables, and a good correlation is considered when the coefficient is ≥ 0.70, with statistical significance for *p* < 0.05.^25^

## Results

The first stage included data from the application of the questionnaire in 20 patients. Of these, 12 were female and 8 were male, with a mean age of 55 ± 22 years. There were no difficulties in understanding any of the sentences in the questionnaires (Table 1).

**Table 1:** Brazilian version of the Tinnitus Primary Function Questionnaire (TPFQ).

1	Eu tenho dificuldade em focar minha atenção em algumas tarefas importantes por causa do zumbido.
2	Eu fico acordado à noite por causa do meu zumbido.
3	Eu só queria que o zumbido acabasse. É muito frustrante.
4	Eu tenho dificuldade em dormir de noite por causa do meu zumbido.
5	Quando há muitas coisas acontecendo ao mesmo tempo, o meu zumbido interfere na minha capacidade de prestar atenção no importante.
6	O meu zumbido atrapalha alguns sons de fala.
7	A minha incapacidade de pensar em algo sem interferências é um dos piores efeitos do meu zumbido.
8	Meu zumbido é irritante.
9	Uma das piores coisas sobre o zumbido é o efeito sobre a compreensão da fala, além dos efeitos da perda auditiva.
10	O zumbido, não minha perda auditiva, interfere na apreciação de música e canções.
11	Eu fico cansado durante o dia porque o meu zumbido interrompe o meu sono.
12	Além da perda auditiva, o zumbido interfere na compreensão da fala.
13	Estou deprimido por causa do meu zumbido.
14	Quando eu acordo à noite, o meu zumbido torna difícil voltar a dormir.
15	Um dos piores efeitos do meu zumbido é a interferência na minha paz emocional.
16	Tenho dificuldade em me concentrar enquanto estou lendo em uma sala silenciosa por causa do zumbido.
17	A dificuldade que eu tenho para dormir é um dos piores efeitos do meu zumbido.
18	Estou ansioso por causa do meu zumbido.
19	Os efeitos do zumbido na minha audição são piores do que os efeitos da minha perda auditiva.
20	Eu sinto que o meu zumbido faz com que seja difícil eu me concentrar em algumas tarefas.

**Score**		
Concentration:	(1 + 5 + 7 + 16 + 20) ÷ 5 =	%
Emotional:	(3 + 8 + 13 + 15 + 18) ÷ 5 =	%
Hearing:	(6 + 9 + 10 + 12 + 19) ÷ 5 =	%
Sleep:	(2 + 4 + 11 + 14 + 17) ÷ 5 =	%
Total:	[Concentration% + Emotional% + Hearing% + Sleep%] ÷ 4 =	%

Table 2 shows that the scores for behavioral aspects and the total score had strong agreement between test and retest. An ICC > 0.82 was observed in all scores (total and by aspect of the questionnaire). For the total score, the ICC was 0.93, which reinforces that the reproducibility and accuracy of the data provided in the TPFQ questionnaire are robust. The TPFQ had a Cronbach’s alpha of 0.95 in the total score, both in the test and in the retest, indicating good internal consistency of its data.

**Table 2:** Interclass correlation coefficients for the TPFQ aspects and total score.

Aspects	ICC
Concentration	0.88
Emotional	0.88
Hearing	0.82
Sleep	0.90
Total Score	0.93

ICC, Interclass Correlation Coefficient.

To validate the TPFQ, data from 1,095 patients seen from September 2019 to March 2021, who underwent tinnitus assessment with the TPFQ and THI in the same consultation, were analyzed, of which 46% were female. The patients came from several Brazilian states, predominantly from the south and southeast regions (Table 3). The internal consistency of the TPFQ and THI was analyzed to verify the homogeneity of the items that comprise the questionnaires. It was considered the total score of both questionnaires and by aspect/domain evaluated (Table 4). The internal consistency was high in the total scores, in all aspects of the TPFQ and in the Functional and Emotional domains of the THI. The Catastrophic domain of THI had a Cronbach’s alpha <0.70, which is the minimum value considered to be consistent.^25^, ^37^

**Table 3:** Demographic characteristics.

Variables	n = 1.095
Age (years)	
Mean ± SD	58.1 ± 14.5
Minimum–Maximum	17–92
Female sex, n (%)	504 (46.0)
State, n (%)	
Rio de Janeiro	275 (25.1)
São Paulo	202 (18.4)
Santa Catarina	187 (17.1)
Rio Grande do Sul	171 (15.6)
Paraná	127 (11.6)
Distrito Federal	73 (6.7)
Minas Gerais	26 (2.4)
Mato Grosso	12 (1.1)
Mato Grosso do Sul	12 (1.1)
Goiás	6 (0.5)
Acre	1 (0.1)
Bahia	1 (0.1)
Pernambuco	1 (0.1)
Tocantins	1 (0.1)

n, number of subjects; SD, Standard Deviation.

**Table 4:** Internal consistency (α) for the Tinnitus Primary Function Questionnaire (TPFQ) and Tinnitus Handicap Inventory (THI) questionnaires.

Questionnaire	⍺
TPFQ	
Total	0.94
Concentration	0.88
Emotional	0.86
Hearing	0.84
Sleep	0.93
THI	
Total	0.93
Catastrophic	0.69
Functional	0.86
Emotional	0.89

⍺, Cronbach's alpha.

**Figure 2:**
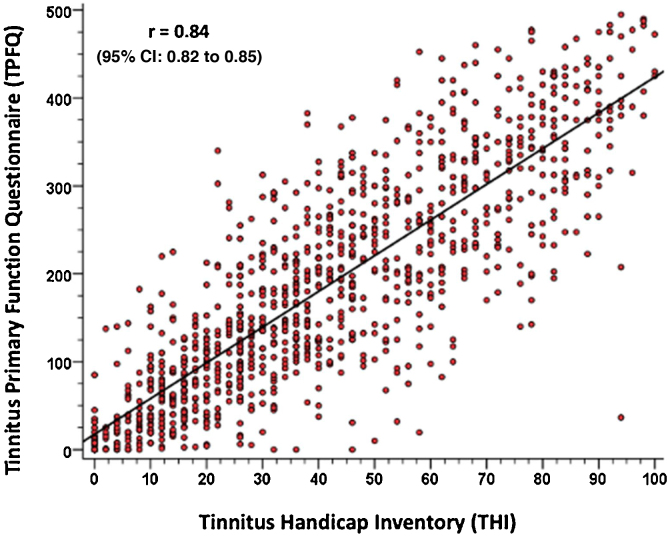
Correlation of the total scores of the TPFQ and THI questionnaires.

To validate the TPFQ, Pearson’s correlation coefficient (r) was calculated by comparing the total scores of both questionnaires. Fig. 2 shows the strong correlation found between both questionnaires (*r* = 0.84, with a confidence interval of 0.95%). It is observed that the higher the TPFQ score, the higher the THI score (*r* = 0.84; *p* < 0.001; 95% CI 0.82–0.85).

In view of the strong correlation found between the data, a linear regression equation is proposed, which allows predicting the result of the THI questionnaire, according to the result of the total score of the TPFQ questionnaire, as described below: Total THI Score = 9.482 + (0.172  × Total TPFQ Score).

Knowing that the THI is widely used in the Brazilian scientific and clinical environment, the correlation between the four aspects of the TPFQ and the THI domains was performed to assist in the clinical interpretation of the two questionnaires (Table 5). Table 5 shows a strong positive correlation between the subdivisions of the questionnaires, especially between the Functional domain of the THI and the Concentration and Emotion aspects of the TPFQ; and, between the Emotional domain of the THI and the Emotion aspect of the TPFQ, for 1,095 patients.

**Table 5:** Correlation between the aspects and domain of the Tinnitus Primary Function Questionnaire (TPFQ) and Tinnitus Handicap Inventory (THI) questionnaires.

Questionnaire		THI Catastrophic	THI Functional	THI Emotional
THI Catastrophic	*r*	1		
	*p*			
THI Functional	*r*	0.65*	1	
	*p*	<0.001		
THI Emotional	*r*	0.74*	0.77*	1
	*p*	<0.001	<0.001	
TPFQ Concentration	*r*	0.54*	0.80*	0.63*
	*p*	<0.001	<0.001	<0.001
TPFQ Emotional	*r*	0.68*	0.71*	0.81*
	*p*	<0.001	<0.001	<0.001
TPFQ Haring	*r*	0.41*	0.62*	0.46*
	*p*	<0.001	<0.001	<0.001
TPFQ Sleep	*r*	0.47*	0.63*	0.53*
	*p*	<0.001	<0.001	<0.001

TPFQ, Tinnitus Primary Function Questionnaire; THI, Tinnitus Handicap Inventory; *p*, *p*-value; significance test (a measure of the probability that an observed difference could have occurred only by chance); r, Pearson’s correlation coefficient.

* Correlation with significance at the 0.01 level (two-tailed).

## Discussion

The application of questionnaires has been widely used in clinical practice for the care of patients with tinnitus.^38^, ^39^, ^40^, ^41^ However, the secure use of a questionnaire depends on its translation, cultural adaptation and validation in the native language.^41^, ^42^, ^43^ It is essential to test the questionnaire among native-speaking citizens, as well as verify its reliability and validity.^28^, ^29^, ^37^, ^44^

The aim of this study was to translate, culturally adapt and validate the TPFQ questionnaire in Brazilian Portuguese, so that it can be used in research and clinical practice. This study involved a significant number of patients (n = 1,095) aged between 17 and 92 years, from 14 different states in Brazil, that is, a geographically significant sample of the Brazilian population. The results of the present study clearly show that the TPFQ questionnaire shows adequate reproducibility in the test-retest in the selected sample and disclose an ICC > 0.82 in all evaluated aspects and in the total score. The translation of this same instrument into the Korean language, for instance, showed ICC similar to that found in the present study (between 0.50 and 0.82, in the sentences).^21^ The translation of the short version of the questionnaire into Mandarin also showed a similar ICC, with the exception that the emotional aspect showed an ICC < 0.70 (0.86 concentration; 0.61 emotional; 0.89 hearing; 0.76 sleep; and 0.85 in the total score).^20^ The finding by Lu et al.^20^ may be related to the difference in the number of sentences contained in the short version, when compared to the full questionnaire. The full questionnaire used in the present study, consisting of 20 sentences, allocates five for each aspect among the four evaluated ones; while the short version has only 12 sentences, that is, three for each aspect.

The reliability of the TPFQ in Brazilian Portuguese was verified by calculating Cronbach’s alpha, which was high for the total score (0.94) and for the aspects (concentration: 0.88, emotion: 0.86, hearing: 0.84 and sleep: 0.93) of the questionnaire. These findings are in agreement with the original validation,^29^ which showed a Cronbach’s alpha of 0.92 for the total score when applying the TPFQ and a variation from 0.81 to 0.94 for the aspects: 0.88 for concentration; 0.84 for emotion; 0.81 for hearing; and 0.94 for sleep. Studies that validated the TPFQ in other languages ​​also showed similar reliability, ranging from 0.82 to 0.95 for the total score.^20^, ^21^, ^22^, ^24^, ^29^

Another relevant issue that should be mentioned is the type of validation used in this study. The validity of the questionnaire can be assessed using different approaches. The present study used the “criterion validity”, which correlates the instrument to be validated (the TPFQ questionnaire, in this case) with another widely accepted questionnaire. Therefore, the THI was chosen, because it is a widely studied and validated questionnaire, used in and translated into several languages, aiming to assess the severity of tinnitus.^14^, ^25^

In the validation of the TPFQ questionnaire, a strong correlation was found between the findings of the two instruments (TPFQ and THI), as shown in Fig. 2, that is, the higher the THI total score, the higher the TPFQ total score. In the literature, the only study that allowed validating the TPFQ, when comparing it with the THI questionnaire, was the study by Lu et al.^20^ In it, the TPFQ was validated for Mandarin (Chinese language variant). A correlation coefficient of 0.70 was found between the total scores of the two instruments. This value is considered valid, but below the one found by the present study. This can be attributed to the difference between the versions validated in the studies. In the study by Lu et al.,^20^ the short version of the TPFQ (12 sentences) was validated and in the present study, the full version (20 sentences) was used.^19^, ^20^

The sample size was more significant (n = 1,095) in the present study when compared with validations made for other languages, in which the maximum number of patients was 350.^20^, ^21^, ^22^, ^24^, ^29^

The results of the TPFQ and THI questionnaires show a strong correlation and a high level of significance. The correlation observed for the data obtained in the two questionnaires allowed the creation of a linear regression equation, which allows predicting the THI results based on the application of the TPFQ. For instance, if the application of the TPFQ results in a total score of 350, when using the linear regression equation it can be said that the THI, for the same patient, will have a total score of 70. This regression equation helps in clinical practice and in research, as it is often not possible to use more than one questionnaire, or if one wants to compare the results of the TPFQ with the THI, applied at another moment.

The TPFQ also provides insight into aspects of quality of life that are directly affected by tinnitus, which is crucial, as it can guide the clinical approach in educational counseling and rehabilitation. Thus, the therapist can be more assertive in approaching the treatment of tinnitus. Moreover, the analysis between the TPFQ aspects and the THI domains showed an aligned correlation between them, except for the catastrophic domain of THI, which showed moderate and low correspondence with the TPFQ aspects.

## Conclusion

The TPFQ questionnaire was translated and culturally adapted into Brazilian Portuguese. The validation of the TPFQ questionnaire showed high reliability and a strong correlation with the THI, which allowed the creation of a linear regression equation that allows calculating the total THI score from the TPFQ, a facilitator in clinical practice and in future research.

## Other information

This study is a doctoral thesis developed in the Postgraduate Program in Otorhinolaryngology at Medical School of the University of São Paulo.

## Conflicts of interest

The authors declare no conflicts of interest.
